# Changes of Gut Microbiota by Natural mtDNA Variant Differences Augment Susceptibility to Metabolic Disease and Ageing

**DOI:** 10.3390/ijms23031056

**Published:** 2022-01-19

**Authors:** Axel Künstner, Paul Schilf, Hauke Busch, Saleh M. Ibrahim, Misa Hirose

**Affiliations:** 1 Lübeck Institute of Experimental Dermatology, University of Lübeck, 23562 Lübeck, Germany; axel.kuenstner@uni-luebeck.de (A.K.); Paul.Schilf@uksh.de (P.S.); hauke.busch@uni-luebeck.de (H.B.); Saleh.Ibrahim@uksh.de (S.M.I.); 2 Institute for Cardiogenetics, University of Lübeck, 23562 Lübeck, Germany; 3 College of Medicine and Sharjah Institute for Medical Research, University of Sharjah, Sharjah 27272, United Arab Emirates

**Keywords:** mitochondrial DNA polymorphisms, natural variants, gut microbiota, complex I, proteobacteria, glucose metabolism, ageing

## Abstract

We recently reported on two mouse strains carrying different single nucleotide variations in the mitochondrial complex I gene, i.e., B6-mt^BPL^ mice carrying m.11902T>C and B6-mt^ALR^ carrying m.4738C>A. B6-mt^BPL^ mice exhibited a longer lifespan and a lower metabolic disease susceptibility despite mild mitochondrial functional differences in steady-state. As natural polymorphisms in the mitochondrial DNA (mtDNA) are known to be associated with distinct patterns of gut microbial composition, we further investigated the gut microbiota composition in these mice strains. In line with mouse phenotypes, we found a significantly lower abundance of *Proteobacteria*, which is positively associated with pathological conditions, in B6-mt^BPL^ compared to B6-mt^ALR^ mice. A prediction of functional profile of significantly differential bacterial genera between these strains revealed an involvement of glucose metabolism pathways. Whole transcriptome analysis of liver samples from B6-mt^BPL^ and B6-mt^ALR^ mice confirmed these findings. Thus, both host gene expression and gut microbial changes caused by the mtDNA variant differences may contribute to the ageing and metabolic phenotypes observed in these mice strains. Since gut microbiota are easier to modulate, compared with mtDNA variants, identification of such mtDNA variants, specific gut bacterial species and bacterial metabolites may be a potential intervention to modulate common diseases, which are differentially susceptible to individuals with different mtDNA variants.

## 1. Introduction

The mammalian mitochondrial DNA (mtDNA) encodes 37 genes, including 13 protein-coding genes for oxidative phosphorylation (OXPHOS) machinery, 22 transfer RNA genes and 2 ribosomal RNA genes [1]. Some variations (both mutations and polymorphisms) are known to cause dysfunction in mitochondria, such as increased ROS production and reduced OXPHOS activities, and consequently result in pathological conditions including primary mitochondrial diseases [2,3,4,5]. Since mitochondrial function is central for cellular metabolism and activities, such dysfunctions are directly linked to health and diseases other than primary mitochondrial disorders. In fact, studies demonstrating that the association of mtDNA variants with common complex diseases, including ageing and age-associated diseases, have been reported in humans [6], and these are supported by a number of experimental observations using mammalian models, including conplastic mouse strains that carry distinct variants in mtDNA [7,8,9,10,11].

At the same time, such common diseases are reportedly associated with the composition of the gut microbiome [12,13]. Our group and others have revealed previously that mtDNA variants are associated with the composition of the gut microbiome [14,15]. One study demonstrated that differential mitochondrial ROS levels caused by ageing or mtDNA mutations are associated with the abundance of specific bacterial species [15]. Similarly, a mouse model with accelerated ageing due to a knock-in mutation at the proofreading domain of the mtDNA polymerase gamma also had changes in gut microbiota composition in addition to mitochondrial dysfunction [16]. In contrast, we recently reported that two mouse strains that carry two nucleotide single nucleotide variants in the mtDNA-coded mitochondrial complex I gene, i.e., B6-mt^ALR^ mice carry m.4738C>A and B6-mt^BPL^ mice with m.11902T>C, do not exhibit major mitochondrial dysfunction in steady-state [17]. More specifically, the levels of OXPHOS complex enzyme activities and mitochondrial super oxide as well as mitochondrial membrane potentials were comparable between the B6-mt^BPL^ and B6-mt^ALR^ mice, while the levels of maximal respiration and spare capacity exhibited a trend of reduction in B6-mt^ALR^ compared with B6-mt^BPL^ mice [17].

As a follow-up study of our previous findings in B6-mt^BPL^ and B6-mt^ALR^ mice, we now sought to elucidate further consequences of the single nucleotide difference in mtDNA variants in complex I, which putatively influences the lifespan and glucose metabolism, with a specific focus on the gut microbiota.

## 2. Results

### 2.1. Proteobacteria, a Marker for Host Health, were Differently Abundant in B6-mt^BPL^ Mice Compared with B6-mt^ALR^ Mice

The mouse groups used in this study were summarised in Table S1. A total of 13 mice were fed with high-fat diet (HFD) and 6 mice with control diet (CD) in each of B6-mt^BPL^ and B6-mt^ALR^ mice for 8 weeks. Stool samples from these mice were collected before dietary intervention (week 0) and at the end of the experiment (week 8) and on average 10,366 (s.d. ± 3178) contigs were used per sample after processing the data (min: 3899; max: 17,908).

**Figure 1:**
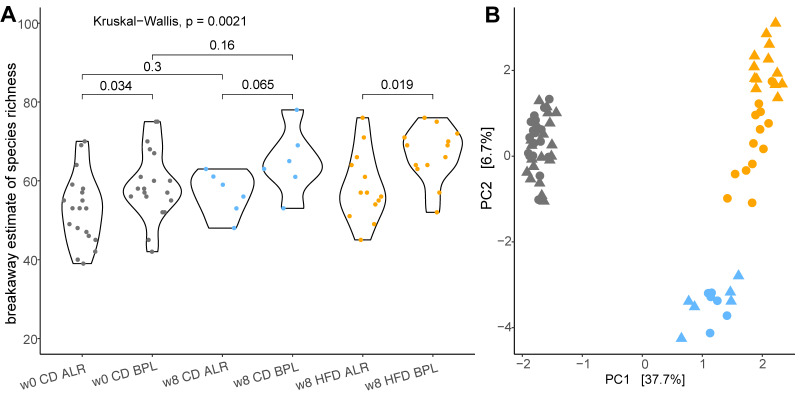
Alpha diversity and beta diversity of gut microbiota in B6-mt^BPL^ and B6-mt^ALR^ mice. (**A**) Alpha diversity plot depicting the breakaway estimate of species richness. Dots denote individual estimates of species richness and violins show the distribution of the data. (**B**) Redundancy analysis plot of beta diversity showing Aitchison distances to assess differences in community composition. Grey refers to mice at week 0 (w0), blue to control diet fed (CD) mice at week 8 (w8) and orange to mice on high-fat diet (HFD), respectively. B6-mt^ALR^ mice are shown in dots and B6-mt^BPL^ mice are shown in triangles. First principal coordinate (PC1) explains 37.7% of the total variation observed, PC2 explains an additional 6.7%.

Bacterial DNA isolated from stool samples were analysed for estimated bacterial richness by alpha diversity, and the difference of the gut bacterial community composition was evaluated by beta diversity. No correlation between sequencing depth and species richness was detected (linear regression: *p* = 0.0756, R^2^_adj_ = 0.0214). Alpha diversity showed a general trend towards higher species richness of gut bacteria in B6-mt^BPL^ mice compared to B6-mt^ALR^ mice; the difference was significant and more prominent when mice were on HFD (*p* = 0.019; Figure 1A). The same was observed for the Shannon index (week 8 B6-mt^BPL^ vs. B6-mt^ALR^: *p* = 0.034, Figure S1). Absolute species turnover, a measure of beta diversity, was estimated using an Aitchison distance matrix and showed significant differences between strains (PERMANOVA, *p* = 0.0007, R^2^ = 0.0355), and time of sampling (week 0 vs. week 8: *p* = 1.0 × 10^−5^, R^2^ = 0.3654) (Figure 1B). Additionally, the interaction of strain and diet was found to be significantly different as well (*p* = 1.0 × 10^−5^, R^2^ = 0.0949).

**Figure 2:**
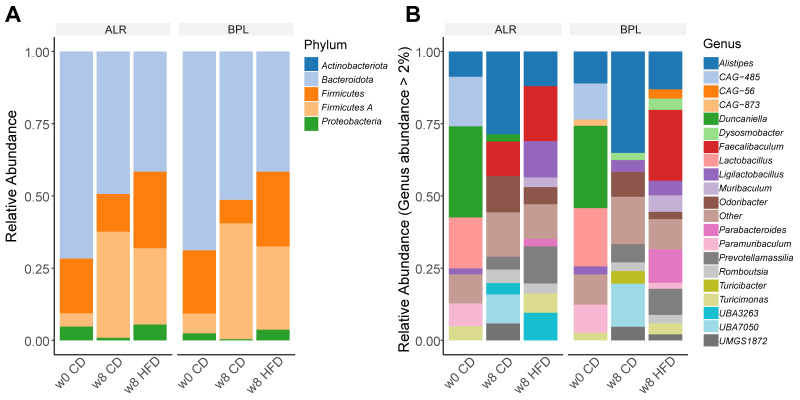
Relative taxonomic abundance comparison between B6-mt^BPL^ and B6-mt^ALR^ mice with different diet groups at the phylum level (**A**) and at the genus level (**B**) BPL = B6-mt^BPL^, ALR = B6-mt^ALR^, w0 = week 0, w8 = week 8, CD = control diet, HFD = high fat diet.

Next, we compared the taxonomic abundances at the phylum and the genus levels for the two strains and differently diet-fed groups (Figure 2, Figure S2, Table 1). At the phylum level, we found that *Proteobacteria* phyla were significantly lower in B6-mt^BPL^ compared to that in B6-mt^ALR^ in all conditions studied (fdr < 0.05), i.e., before the dietary change (week 0; Figure S2A), after 8 weeks of CD feeding (Figure S2B), and after 8 weeks of HFD feeding (Figure S2C). This phylum was significantly increased by HFD in both strains (Figure S2D,E). Upon the HFD feeding, six bacterial genera, namely *Alistipes*, *Duncaniella*, *Odoribacter*, *UMGS1862*, *CAG-873* and *Acutalibacter* were all significantly reduced in abundance in both mouse strains (fdr < 0.05; Table 1). In B6-mt^BPL^ mice, regardless the diet type (i.e., both CD and HFD), one bacterial genus called *UBA3263* (former Porphyromonadaceae bacterium) was significantly less abundant compared to B6-mt^ALR^ mice in the respective diet group (HFD, *p* = 1.1092 × 10^−21^, effect size = −2.0279; CD, *p* = 1.1536 × 10^−08^, effect size = −1.1879). At the same time, the abundance of *UBA3263* was significantly increased in B6-mt^ALR^ mice when they were fed with HFD (*p* = 1.8992 × 10^−10^, effect size 1.5843), albeit this phenomenon was absent in B6-mt^BPL^ (Table 1).

**Figure 3:**
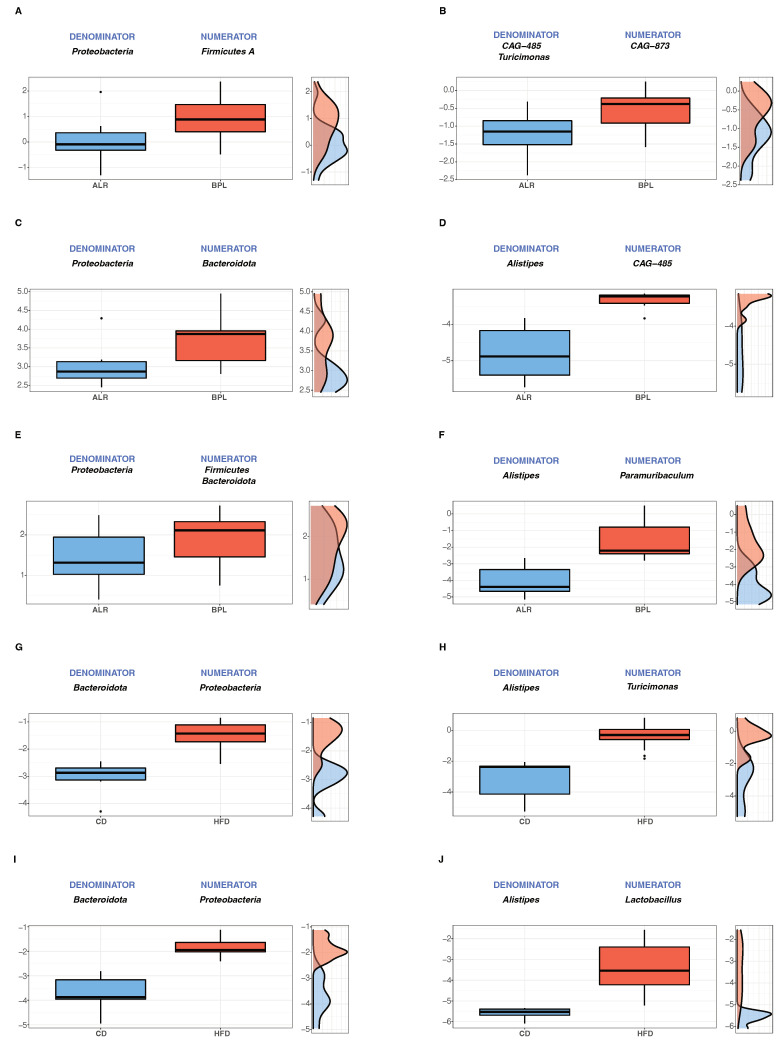
Description of the global balance of bacterial taxa between strains and dietary conditions. The two groups of taxa that form the global balance (defined as denominator and numerator) are specified at the top of the plot. The box plots depict the distribution of the balance score and the density of each distribution is shown next to the box plot for each compared group. The *y*-axis of the plots indicates the balance score. (**A**,**C**,**E**,**G**,**I**) show the analysis at the phylum levels, while (**B**,**D**,**F**,**H**,**J)** are at the genus level. Comparison between B6-mt^BPL^ and B6-mt^ALR^ mice at week 0 (phylum level **A**, genus level **B**), CD-fed for 8 weeks (**C**,**D**), HFD-fed for 8 weeks (**E**,**F**). Comparison between HFD and CD groups in B6-mt^ALR^ (**G**,**H**) and B6-mt^BPL^ (**I**,**J**).

To further analyse microbial signatures in relation to phenotypes and to consider the compositional nature of microbiome data, we calculated balances of bacterial taxa, using a greedy stepwise algorithm (selbal) with 5-fold cross validation. This method estimates the optimal number discriminating taxonomic groups and the ratio of these taxonomic groups (named as ’’denominator’’ and ‘’numerator’’), and then defines the balance between the microbial characteristics that best describe the differences of the compared phenotypes. Before dietary change (week 0), the estimated differential balance of bacteria between B6-mt^ALR^ and B6-mt^BPL^ mice (Figure 3A) resembled the results of the differential abundance analysis, i.e., B6-mt^BPL^ mice have a higher/lower abundance of *Firmicutes A*/*Proteobacteria* than B6-mt^ALR^ (Figure S2A). The discrimination value of this comparison (mean area under the ROC curve; AUC) was 0.809, suggesting a high accuracy of the estimate. For mice CD-fed for 8 weeks, the balance between *Proteobacteria* (assigned as denominator) and *Bacteroidota* (assigned as numerator) was evaluated between B6-mt^BPL^ and B6-mt^ALR^ mice at the phylum level (Figure 3B). An increase in the phylum *Proteobacteria* in B6-mt^ALR^ and a higher abundance of phylum *Bacteroidota* in B6-mt^BPL^ were observed (AUC = 0.778; Figure 3C). At the genus level, *Alistipes* and *CAG-485* were the most discriminating taxa, in B6-mt^ALR^ and B6-mt^BPL^, respectively (AUC = 1.000 Figure 3D). For the HFD-fed mice at week 8, the global balance of the phylum *Proteobacteria* (denominator) and a group of phyla consisting of *Bacteroidota* and *Firmicutes* (numerator) changed towards a higher balance of *Proteobacteria* in B6-mt^ALR^ mice, while the numerator phylum was higher in B6-mt^BPL^ mice (AUC = 0.751; Figure 3E). At the genus level, *Alistipes* (B6-mt^ALR^) and *Paramuribaculum* (B6-mt^BLP^) showed the best discrimination between strains (AUC = 1.000, Figure 3F). When compared with CD-fed and HFD-fed groups, *Bacteroidota* and *Proteobacteria* were the best discriminating phyla for CD-fed group and HFD-fed group, respectively in each strain (B6-mt^ALR^ AUC = 1.000, Figure 3G; B6-mt^BPL^ AUC = 1.000, Figure 3I). At the genus level, *Alistipes* was best discriminating and more abundant compared with respective numerator taxa in CD-fed mice in both B6-mt^ALR^ and B6-mt^BPL^ strains. *Turicimonas* was found to be high B6-mt^ALR^ mice on HFD, whereas *Lactobacillus* was higher abundant in HFD-fed B6-mt^BPL^ (B6-mt^ALR^ AUC = 1.000, Figure 3H; B6-mt^BPL^ AUC = 1, Figure 3J).

### 2.2. Correlation between the Abundance of Commensal Bacteria and Alteration in Metabolic Parameters upon the Dietary Metabolic Stress

To evaluate whether identified differences in the abundance of bacteria were associated with metabolic parameters observed in these mice, we conducted a correlation analysis between metabolic parameters and the abundance of gut bacterial taxa in each individual. The summary of the metabolic phenotype data is presented in Figure S3.

After 8 weeks of dietary stress, glucose metabolism was evaluated by intraperitoneal glucose tolerance test (ipGTT) and body weight was measured in the mice. From the collected data, the fasting glucose values (mmol/L), the glucose levels at 45 min of the ipGTT, and body weight were selected for the correlation analysis. The glucose levels at 45 min of ipGTT was selected for the correlation analysis as this was the time point with the most prominent and significant difference between HFD-fed and CD-fed mice groups (Figure S3A,B).

**Figure 4:**
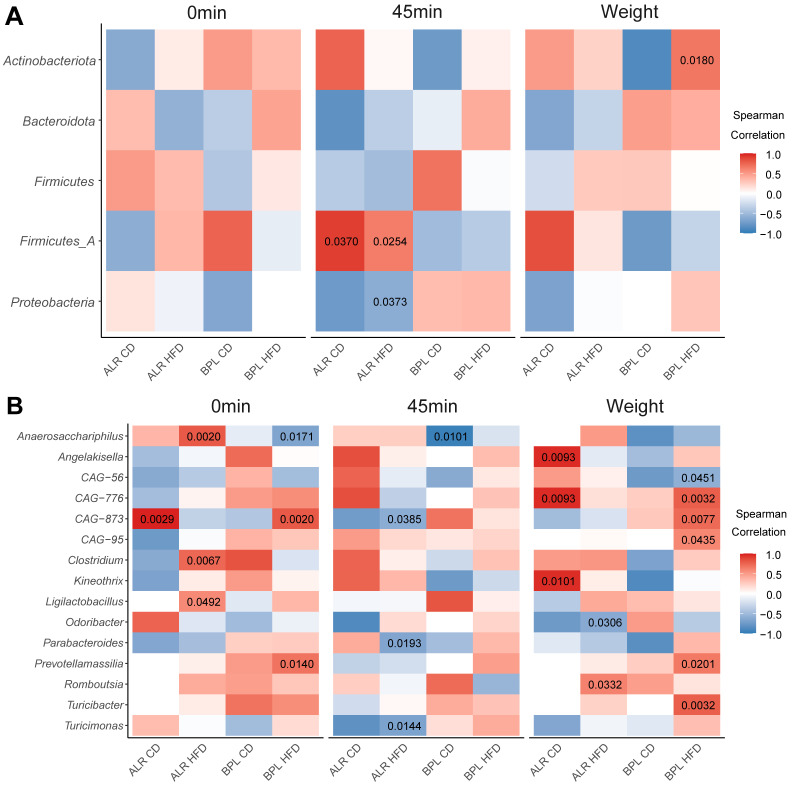
Correlation of bacterial phylum and genus with metabolic parameters. (Panel **A**) shows the correlation with bacterial phyla and (Panel **B**) are those with a genus. Correlation of bacterial abundance with fasting glucose levels (0 min), that with area glucose levels at 45 min after glucose injection in ipGTT (45 min), and that with body weight (Weight). *p*-values smaller than 0.05 are presented in the heat maps; only genera were included where the *p*-value was below 0.05 in at least one comparison.

First, to evaluate whether the abundance of specific bacterial phylum and/or genus correlating with respective metabolic phenotype are commonly shared in all mice, we looked for the similar trends of correlation in all four groups, i.e., bacterial abundance correlation with the similar colours within a bacterial genus in each heat map. There were no bacterial phyla and/or genera that had a similar impact (i.e., negative or positive correlations) in fasting glucose (0 min) levels the glucose levels at 45 min after the glucose injection in ipGTT (Figure 4A).

When we looked for strain-specific and/or non-diet-specific correlations, *Prevotellamassilia* showed significant positive correlations with fasting glucose levels and with body weight in HFD-fed B6-mt^BPL^ mice (Spearman’s rho = 0.6849 and *p* = 0.0140, rho = 0.6579 and *p* = 0.0201, respectively) and weak positive correlations in CD-fed B6-mt^BPL^ mice (rho = 0.5000 and *p* = 0.3910, rho = 0.2500 and *p* = 0.6850, respectively; Figure 4B). A positive, but non-significant correlation of *Prevotellamassilia* with glucose levels at 45 min after glucose injection was also observed in B6-mt^BPL^ mice (HFD-fed, rho = 0.4811 and *p* = 0.1133; CD-fed, rho = 0.0 and *p* = 0.1; Figure 4B) yet this was absent in both HFD-fed nor CD-fed B6-mt^ALR^ mice.

A strong correlation between ipGTT response after 45 min and the phyla *Firmicutes A*, as well as *Proteobacteria*, was identified in CD-fed and HFD-fed B6-mt^ALR^ mice (*Firmicutes A*: CD-fed *p* = 0.0370, HFD-fed *p* = 0.0254, *Proteobacteria*: CD-fed *p* = 0.1417, HFD-fed *p* = 0.0373). These correlations were inversed, but were not significant in the respective groups of the B6-mt^BPL^ mice.

### 2.3. Functional Profiles of Differentially Abundant Gut Bacteria between HFD-fed B6-mt^BPL^ and B6-mt^ALR^ Revealed Significant Involvement of Glucose Metabolism in Gut-Microbially Derived Pathways

**Figure 5:**
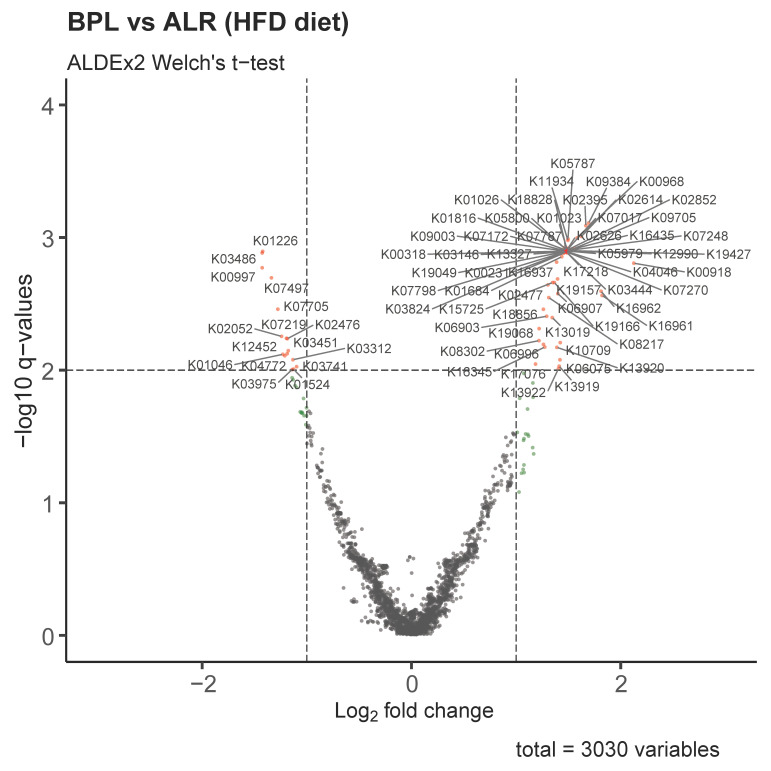
Volcano plot illustrating differentially involved KEGG terms based on the differentially abundant bacterial taxa between HFD-fed B6-mt^ALR^ and B6-mt^BPL^ mice. Each ID denotes respective KEGG ID. Pathways with the absolute log_2_ fold change greater than 1 are considered to have sufficiently large positive and negative effect, respectively, and those with an adjusted p-values of minus log_10_ *q*-value greater than 2 (*q* < 0.01) are regarded as significant. BPL = B6-mt^BPL^, ALR = B6-mt^ALR^.

To evaluate the functional relevance of the differentially abundant bacterial taxa between the two strains upon dietary intervention, we predicted functional profiles using PICRUSt2 and conducted differential abundance analysis of the identified KEGG gene orthologues of the gut microbiota between B6-mt^BPL^ and B6-mt^ALR^ mice fed with HFD over 8 weeks. By this analysis, we observed 16 KEGG gene orthologues (across 9 unique pathways) that were upregulated in HFD-fed B6-mt^ALR^ and 58 KEGG terms (across 32 unique pathways) that were found to be upregulated in HFD-fed B6-mt^BPL^ mice (Figure 5, Table S2). Of the latter 58 KEGG gene orthologues, the ADP-dependent phosphofructokinase/glucokinase (K00918) was most positively involved in HFD-fed B6-mt^BPL^ mice (effect size 2.1382; *q* = 0.0013). Among the 15 KEGG gene orthologues upregulated in HFD-fed B6-mt^ALR^ mice, triacylglycerol lipase (K01046), which is involved in glycerolipid metabolism, was significantly altered (effect size −1.2237; *q* value = 0.0075).

We next revisited our previously published liver transcriptomics data of intact B6-mt^BPL^ and B6-mt^ALR^ mice in the search for relevant pathways and cellular processes associated with the differentially abundant bacteria profiles [17]. In B6-mt^BPL^ mice, biological processes, cellular components, and molecular functions, including response to insulin (*p* = 0.0019), hexose and glucose transmembrane transport (both *p* value = 0.0050), cellular response to insulin stimulus (*p* = 0.0086), regulation of insulin secretion involved in cellular response to glucose-to-glucose stimulus (*p* = 0.0090), and insulin receptor substrate binding (*p* = 0.0135) were upregulated compared with B6-mt^ALR^ mice. Additionally, ATP-binding cassette (ABC) transporter genes, particularly the lipid transporters, i.e., Abca6, and Abca8b, were significantly upregulated in B6-mt^BPL^ compared with B6-mt^ALR^ (*p* = 2.989 × 10^−6^ and 0.0016, respectively).

## 3. Discussion

The impact of natural variations of mtDNA on various health and disease phenotypes, including lifespan and chronic inflammatory disease susceptibility has been explored [8,9,11,17,18]. Regardless of the position of the mtDNA variants, the functional consequences of such variants (e.g., mitochondrial functions, such as ATP production, mitochondrial ROS production, and mitochondrial OXPHOS enzymatic activities) were very mild in steady-state, particularly when compared to the effects of the classical deleterious mtDNA mutations. Despite these facts, mice carrying such natural mtDNA variants consistently showed clear phenotypic differences compared with their respective genetic control/reference strains [8,9,11,17].

One of the potential contributions of such mtDNA variants to the effects on observed phenotypes involves interactions with gut microbiota. Previously, our group and others demonstrated that pathological mutations or natural variants in mtDNA are associated with distinct patterns of the gut microbiota [14,15]. One report demonstrated that the mitochondrial ROS levels correlated with the diversity of the gut microbiota, in other words, mtDNA genotypes, which clearly showed increased mitochondrial ROS levels, were associated with less diversity of the gut microbiota composition [15]. Here, we focused on the analysis of mtDNA variants that cause only mild mitochondrial functional differences.

Recently, we reported that single nucleotide variant differences of mtDNA, natural polymorphisms in mitochondrial complex I, were associated with lifespan differences and differential response to metabolic stress, when comparing B6-mt^BPL^ and B6-mt^ALR^ mice. Since mitochondrial functional differences were minor between mice carrying the natural mtDNA variants in unchallenged or unstressed conditions, we induced diet stress and analysed the ensuing gut microbiota composition in the B6-mt^BPL^ and B6-mt^ALR^ mice. In B6-mt^BPL^, the abundance of one bacterial phylum, *Proteobacteria,* was significantly less compared to B6-mt^ALR^ mice, when the mice were fed with CD, as well as with HFD. Upon the HFD feeding, we observed higher abundance of *Proteobacteria* compared with CD-fed groups in both strains, suggesting that this bacterial phylum may be an indicator for diet-induced obesity. Interestingly, a Proteobacterial load is known to be positively associated with metabolic disorders [19] and ageing [20]. This is in line with our phenotype observation in B6-mt^BPL^ mice, i.e., more resistant to metabolic stress and a longer lifespan, when compared with B6-mt^ALR^ mice. We have previously demonstrated that this natural mtDNA variant difference results in lower levels of tryptophan (Trp) in the B6-mt^BPL^ compared to the B6-mt^ALR^ variant. Interestingly, *Proteobacteria* and four other phyla have been predicted to have a higher potential to metabolise Trp [21]. Hence, the single mtDNA variant difference putatively causes a Trp-diminished environment for the gut microbiota. The specific bacteria taxa that rely less on exogenous Trp thus thrive, while taxa that require supplementation of Trp may struggle to maintain their position in the gut microbiota. Yet, experimental confirmation is required to prove this phenomenon.

*UBA3263* (*Porphyromonadaceae bacterium UBA3263*), which was found to be less abundant in B6-mt^BPL^ mice than B6-mt^ALR^ mice, regardless of diet type, showed even more increased abundance in B6-mt^ALR^ mice under HFD conditions. This taxon is reportedly negatively correlated with glucose metabolism [22].

When we conducted the correlation analysis between bacterial abundance and metabolic parameters, we observed that the abundance of *Prevotellamassilia* was significantly positively correlated with the fasting glucose levels and body weight exclusively in HFD-fed B6-mt^BPL^ mice. While no significant differences in abundance of *Prevotellamassilia* was observed between the strains, the selective correlations in HFD-fed B6-mt^BPL^ mice may point towards an interaction of mtDNA variants and gut microbiota affecting the observed changes in metabolic parameters in these mice.

Furthermore, predictive analysis of the functional profiles of the bacteria exhibited differential abundances of microbiota between the two mouse strains. This analysis predicts orthologues gene identifiers (K numbers, e.g., K00918 and K16961) from the differential abundances found in the bacterial sequencing data (ASV). It further allows the prediction of differentially regulated pathways based on the bacterial abundance between the compared groups. In B6-mt^BPL^ mice, 58 bacterially encoded genes and 32 potential pathways were upregulated, while 16 bacterially encoded genes and 9 pathways were downregulated when compared with B6-mt^ALR^ mice under HFD stress. Among the identified upregulated genes in B6-mt^BPL^ mice, the ADP-dependent phosphofructokinase/glucokinase gene was the most abundant. Accordingly, glucose metabolism (i.e., glycolysis and gluconeogenesis) was shown to be the most upregulated, hinting at a potential impact on glucose metabolism in the gut of these mice. Meanwhile, we revisited our previously reported whole transcriptomics data of liver samples from intact B6-mt^BPL^ and B6-mt^ALR^ mice and found that cellular processes such as insulin response and glucose/hexose metabolism were significantly upregulated in B6-mt^BPL^ mice compared to B6-mt^ALR^ mice. Importantly, both intact B6-mt^BPL^ and B6-mt^ALR^ mice do not present any metabolic dysfunction and disorders under the normal housing condition, despite their differential profiles in the transcriptomics analysis. Therefore, the secondary metabolic stress, in our case HFD feeding, augmented the host glucose metabolism by modulating the gut microbial composition to become resistant to metabolic stress in B6-mt^BPL^ mice, but not in B6-mt^ALR^ mice. This may be the same for ageing stress, i.e., functional changes in gut microbiota in aged B6-mt^BPL^ may have a protective effect on the lifespan of B6-mt^BPL^ mice.

Taken together, we provide the first evidence explaining the impact of a single mtDNA natural variant on both host and their intestinal microbial environment, and how these bi-directional changes synergistically alter susceptibility to metabolic and age-related stress, without inducing major changes in mitochondrial functions, e.g., OXPHOS complex activity or mitochondrial reactive oxygen species production, at steady-state. Further studies to identify bacterial species/communities and related bacterial metabolites in individuals with specific mtDNA variants will be required to strengthen the postulated correlation of mtDNA variant-associated common diseases and develop novel therapeutic interventions.

## 4. Materials and Methods

### 4.1. Mice, and Stool Sample Collection

Conplastic mouse strains C57BL-mt^ALR/LtJ^ and C57BL/6J-mt^BPL/1J^ were generated previously [23]. The detail of these mouse strains and that of high-fat diet feeding experiment are described elsewhere [17]. The mutations in mtDNA of each conplastic mouse strain are presented in Table S3. Three to five mice were housed together in each strain, i.e., male HFD groups, n = 5/cage; female HFD groups, n = 4/cage; male and female CD groups, n = 3/cage. Faecal samples were collected from before feeding experiment starts (week 0), and after 8 weeks diet feeding (week 8). Collected faecal samples were stored at −80 °C until further analysis.

### 4.2. Bacterial DNA Isolation and Library Preparation and Sequencing for the Bacterial 16S Ribosomal RNA Gene

Bacterial DNA isolation, library preparation and sequencing for the 16S rRNA gene were conducted as previously described [14]. In brief, bacterial DNA was prepared from the faecal samples using a Power Soil DNA Isolation Kit (Qiagen, Hilden, Germany), according to the manufacturer’s instruction. The hypervariable V1-V2 region of the bacterial 16S rRNA gene was amplified by polymerase chain reaction using the 27F/388R primer combination, employing a dual-index strategy. The PCR products were pooled into equimolar subpools, and followed by purification by magnetic beads. The quality of the final library was determined by Agilent 2100 Bioanalyzer, and the quantity was measured by Qubit. The final library was sequenced on the Illumina MiSeq platform using v3 chemistry (600 cycles, Illumina Inc. San Diego, CA, USA).

### 4.3. Data Process and Analysis

Raw sequence data in *fastq* format were de-multiplexed, and processed into amplicon sequence variants (ASVs), using DADA2 (v1.20.0) [24]. In brief, the expected error rate was assigned the value 2 for forward reads and the value 3 for reverse reads, which resulted in the selection of a minimum read length of 200 bp. Merged sequences (contigs) were selected between 300 bp and 342 bp. Additionally, chimeric sequences were removed following the DADA2 recommendations. For taxonomic assignment, IdTaxa (DECIPHER package (v2.18.1) [25] with GTDB r202 [26] was used as the reference database. Potential contaminants (e.g., bacteria which do not exist in the gut) were removed, using the frequency and prevalence method as implemented in the R package decontam (v1.10.0) [27], with the threshold set to 0.3 for the frequency method and to 0.5 for the prevalence method, respectively. Eight ASVs were identified as contaminants by the frequency method and were excluded. No additional contaminants were identified by the prevalence method. ASVs not belonging to the kingdom Bacteria or with unassigned phylum, were excluded from further analysis.

### 4.4. Statistical Analysis

ASV data and covariates were imported into R (v4.0.3) for further analysis. Alpha diversity was calculated using the species richness estimator as implemented in breakaway (v4.7.3) [28]. Additionally, sample-wise Shannon index was calculated as implemented in the DivNet package (v0.3.7) [29]. Differences in alpha diversity were assessed using nonparametric Kruskal–Wallis test and pairwise Wilcoxon test as a post-hoc test. Beta diversity was estimated using Aitchison distance [30] and permutational multivariate analysis of variance using distance matrices (PERMANOVA) was used to analyse differences in beta diversity (adonis function, vegan package v2.5-7, with 99,999 permutations). To investigate differential abundant taxa, using a beta-binomial regression as implemented in corncob (v0.2.0) [31] with gender in the null model to correct for gender differences. Additionally, balances [32] were calculated using a forward-selection method with 5-fold cross-validation (10 iterations) for the identification of two groups of taxa whose relative abundance (balance) is associated with strains or diet (selbal package v0.1.0). Balances were calculated on the phylum/genus level using only taxa present in at least 25% of the samples and sex was used as a covariate to adjust for sex effects. Partial correlations (Spearman correlation) of *clr* transformed abundance values were calculated using the R package ppcor v1.1 [33] while controlling for sex effects. On the genus level, only genera found in at least 1/6th of the samples were included to calculate partial correlations.

Functional profiles were predicted for the ASV data using PICRUSt2 v2.4.1 [34] with a NSTI cut-off of 0.3, the minimal number of reads set to 20 and the minimal number of samples set to 5. Differential abundant KEGG terms were identified using ALDEx2 v1.24.0 [35] (Welch’s *t*-test *p*-values were corrected for multiple testing using Benjamini–Hochberg correction) and results were visualized using the EnhancedVolcano package v1.10.0.

## Figures and Tables

**Table 1:** The list of differential bacterial taxa at the genus levels in comparison between B6-mt^BPL^ and B6-mt^ALR^ and those with different diet groups.

Comparison	HFD vs. CD in ALR			HFD vs. CD in BPL			BPL vs. ALR in CD			BPL vs. ALR in HFD		
Abundance	Taxa	Fdr	Effect	Taxa	Fdr	Effect	Taxa	Fdr	Effect	Taxa	Fdr	Effect
Decreased	*CAG-269*	0.00005	−4.72158	*Clostridium*	0.01590	−3.29675	*UBA3263*	0.00000	−1.18785	*UBA3263*	0.00000	−2.02792
	*Duncaniella*	0.00018	−3.28685	*Duncaniella*	0.00160	−3.22098	*Duncaniella*	0.00384	−1.02234	*Emergencia*	0.00076	−1.14646
	*CAG-873*	0.00120	−2.87259	*Acutalibacter*	0.00000	−2.29577	*Romboutsia*	0.00021	−0.75258	*Ligilactobacillus*	0.00133	−0.89182
	*Schaedlerella*	0.01477	−2.23013	*Alistipes*	0.00000	−1.65379				*Turicimonas*	0.00317	−0.61016
	*UMGS1872*	0.00119	−1.88033	*CAG-873*	0.00000	−1.28945						
	*Acutalibacter*	0.00003	−1.44531	*Odoribacter*	0.01590	−1.23079						
	*Alistipes*	0.00000	−1.27705	*Anaerosacchariphilus*	0.00001	−1.20352						
	*Odoribacter*	0.02196	−0.91225	*UMGS1872*	0.00019	−1.12353						
	*Romboutsia*	0.00000	−0.53529									
Increased	*UBA3263*	0.00000	1.58432	*Muribaculum*	0.02202	1.00961	*Acutalibacter*	0.04802	0.43005	*Muribaculum*	0.03175	0.82913
	*Ligilactobacillus*	0.00000	2.28024	*Parabacteroides*	0.00000	1.97159	*Bacteroides*	0.00000	0.58530	*Dysosmobacter*	0.00133	1.43358
	*Emergencia*	0.04810	3.84358	*Turicimonas*	0.00000	2.75714	*Turicibacter*	0.00073	0.81148	*Parabacteroides*	0.00000	1.59726
				*Faecalibaculum*	0.00001	3.14495	*Ligilactobacillus*	0.04507	1.08915	*CAG-873*	0.04156	1.98442
				*CAG-56*	0.00118	5.71160	*Faecalibaculum*	0.01479	1.12808	*Paramuribaculum*	0.00533	3.61420
							*CAG-485*	0.01479	1.69376			

## Data Availability

16S rRNA gene sequencing data used for this study were submitted to the European Nucleotide Archive (ENA) and are available under accession number PRJEB48909. A repository with all analysis used in this study is available at https://github.com/kunstner/2021_BPLxHFD_paper (accessed on 13 December 2021).

## Supplementary Materials

The following supporting information can be downloaded at: https://www.mdpi.com/article/10.3390/ijms23031056/s1.
