# Supplementary material for: Changes of Gut Microbiota by Natural mtDNA Variant Differences Augment Susceptibility to Metabolic Disease and Ageing
Source: Int J Mol Sci. 2022 Jan 19;23(3):1056. doi: 10.3390/ijms23031056 (PMC8835372; doi:10.3390/ijms23031056)
Supplement: Supplementary file 1 [file ijms-23-01056-s001.zip › ijms-153257_suppl_proof/ijms-1532574_Suppl_proof.pdf]

Supplementary Materials

Table S1. The list of mouse samples used in the gut microbiota analysis.

	Week 0		Week 8			
	(Before Dietary Intervention)		CD		HFD	
**Strain**	**Male**	**Female**	**Male**	**Female**	**Male**	**Female**
B6-mt^{ALR}	8	11	3	3	5	8
B6-mt^{BPL}	8	11	3	3	5	8

Table S2. The list of KEGG pathways involved in differently abundant bacteria between B6-mt^{BPL} and B6-mt^{ALR} mice.

Separate excel file.

Table S3. The list of mutations in the mtDNA in B6-mt^{BPL} and B6-mt^{ALR} mice.

Position *	4738	11,902
Strain | Gene	*mt-Nd2*	*mt-Nd5*
C57BL/6J-mt$^{BPL/1J}$	C	C
C57BL/6J-mt$^{ALR/LtJ}$	A	T
AA change	Leu-Met	Phe-Leu

* Each mutation is unique for respective conplastic mouse strain, and not observed in other mouse strains to date.

Figure S1

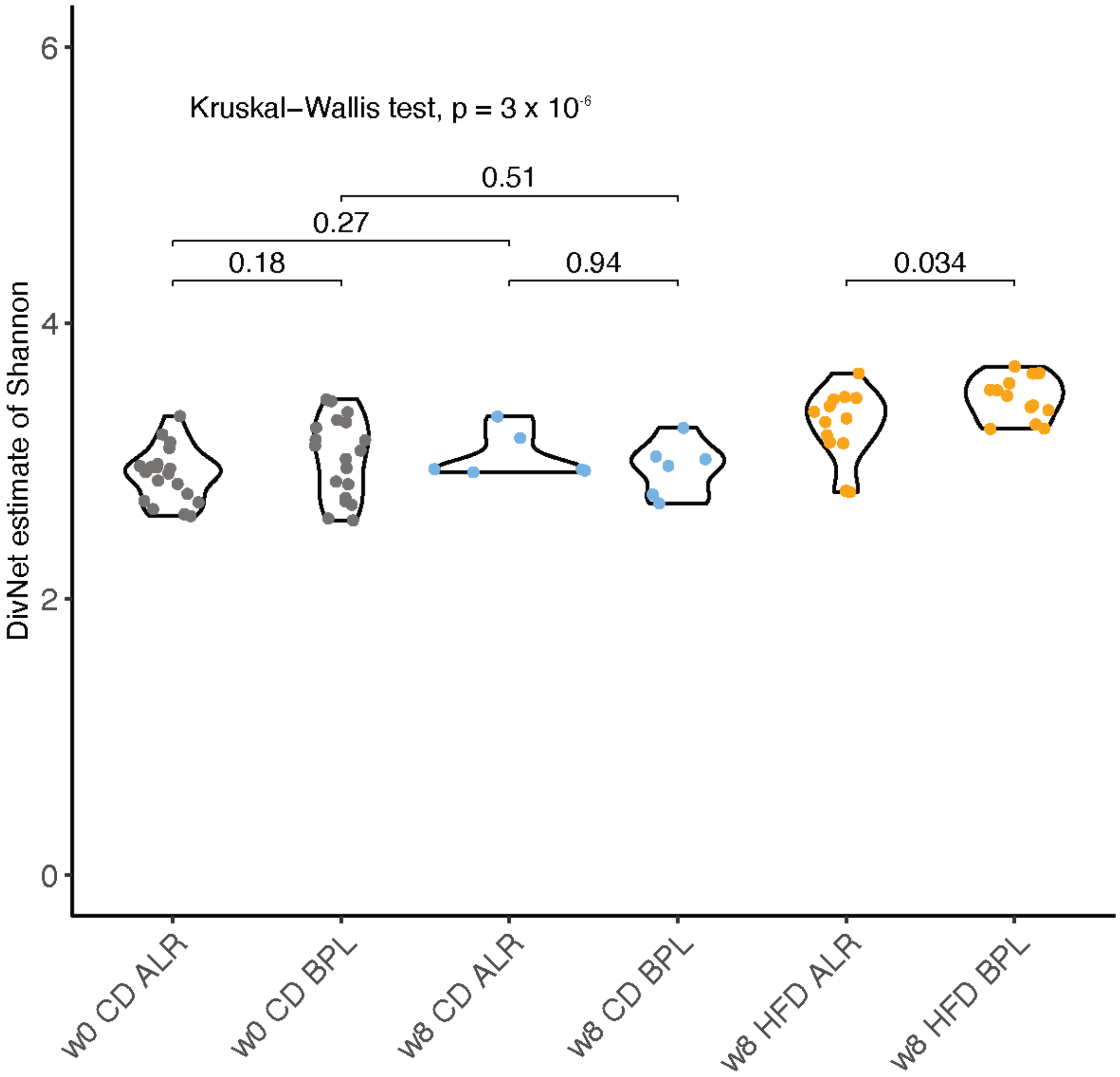

Figure S1: Data related to Figure 1. Alpha diversity plot depicting an estimate of Shannon. w0: week 0, w8: week 8, CD: control diet, HFD: high-fat diet, ALR: B6-mt^{ALR}, BPL: B6-mt^{BPL}.

Figure S2

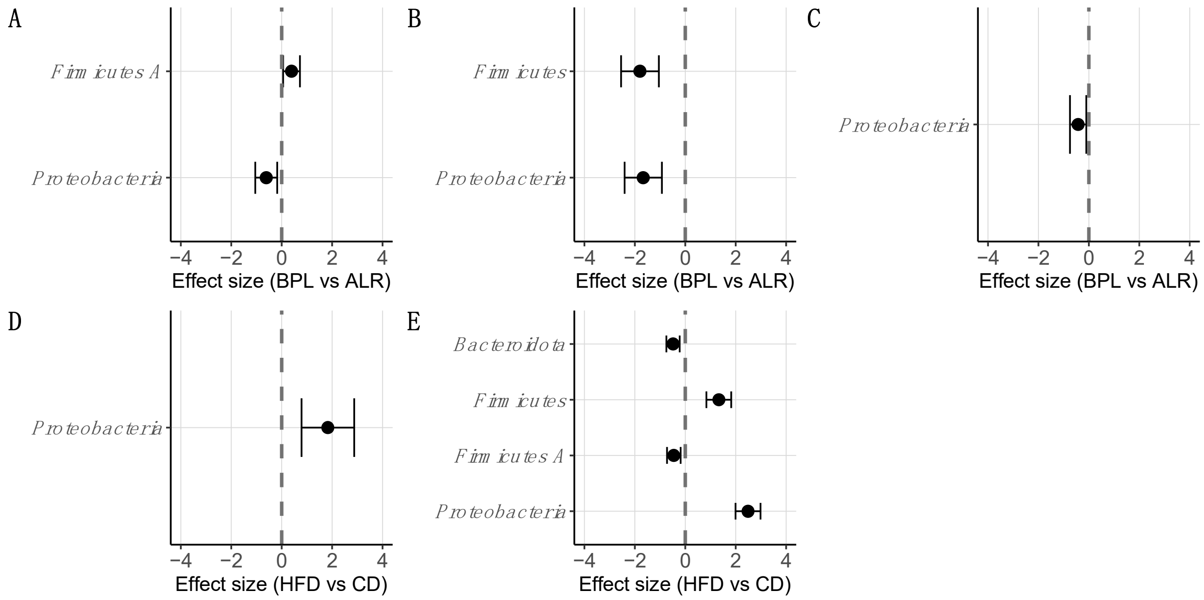

Figure S2: Data related to Figure 2. Bacterial phyla, which were significantly differentially abundant between B6-mt^{BPL} and B6-mt^{ALR} before dietary intervention (week 0; (**A**)), at 8 weeks after CD feeding (**B**), and at 8 weeks after HFD feeding (**C**) are presented. Similarly, those between HFD-fed group and CD-fed group in B6-mt^{ALR} mice (**D**) and in B6-mt^{BPL} mice (**E**) are shown.

Figure S3

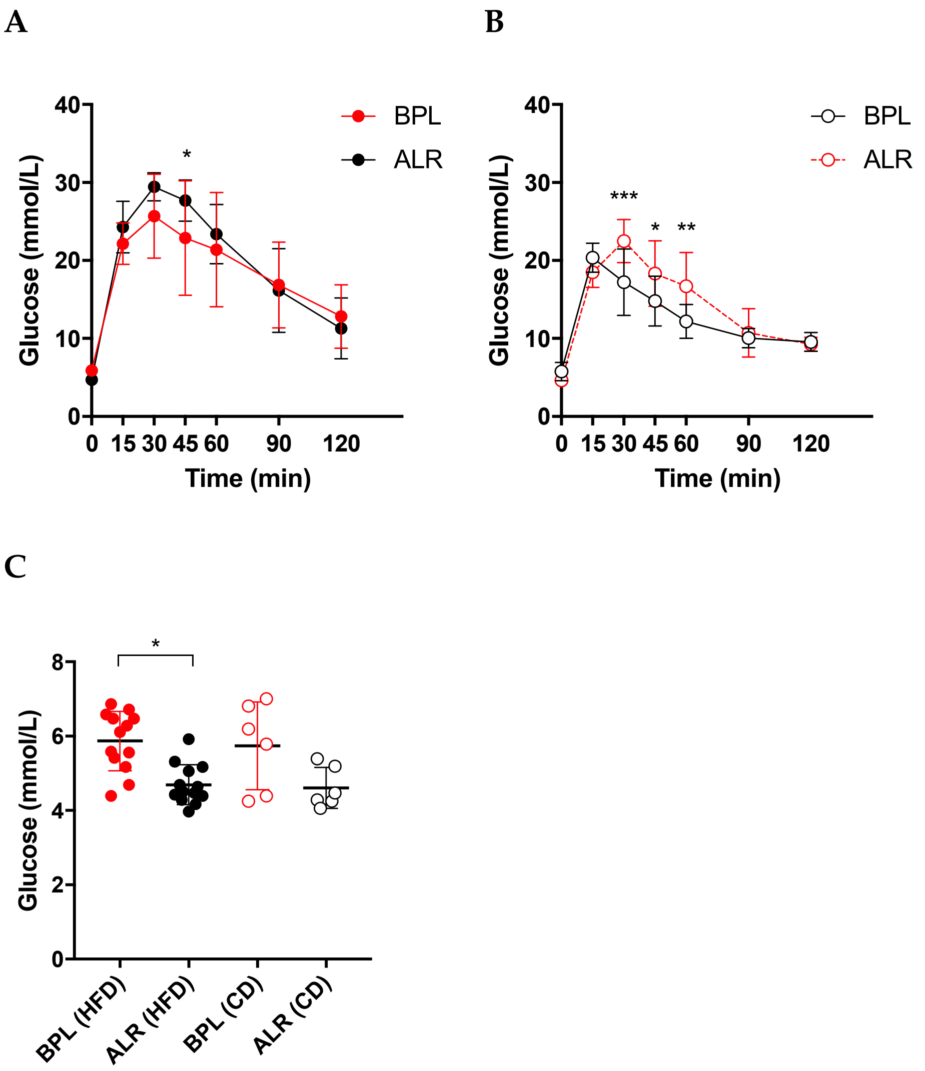

Figure S3: Metabolic phenotype observed in B6-mt^{BPL} and B6-mt^{ALR} mice published in reference 16. (**A**). ipGTT test conducted in HFD-fed mice. BPL = B6-mt^{BPL}, ALR = B6-mt^{ALR}, n = 13 (5 males and 8 females)/strain. * p = 0.0411, two-way ANOVA. (**B**). ipGTT test in CD-fed mice. N = 6 (3 males and 3 females)/strain. * p = 0.0223, ** p = 0.0022, *** p = 0.0003, two-way ANOVA. (**C**). Fasting glucose levels taken in (**A**,**B**). * p = 0.0120, Kruskal-Wallis test. All data is consisted with males and females.
